# Heterotopic pregnancy following ovulation induction by Clomiphene and a healthy live birth: a case report

**DOI:** 10.1186/1752-1947-2-390

**Published:** 2008-12-17

**Authors:** Abbas Honarbakhsh, Elham Khoori, Simin Mousavi

**Affiliations:** 1 Department of Radiology and Ultrasonography, Madaen Hospital, Tehran, Iran; 2 Department of Midwifery, Golestan University of Medical Sciences, PO Box 49165-568, Gorgan, Iran; 3 Department of Obstetrics and Gynaecology, Madaen Hospital, Tehran, Iran

## Abstract

**Introduction:**

A heterotopic pregnancy is defined as the presence of a combined intrauterine and ectopic pregnancy. Its estimated incidence is accepted as between 1/7000 and 1/30,000 pregnancies. It is also reported to be as high as 1% after the use of assisted reproductive technology, but Clomiphene Citrate which increases the rate of twinning, could be associated with a heterotopic pregnancy rate of 1/900, which is much less than using assisted reproductive technology. Heterotopic pregnancies are diagnostic and therapeutic challenges for obstetricians. If they continue without diagnosis, a life-threatening situation may occur even when surgical intervention with laparotomy is performed.

**Case presentation:**

We present the case of a 22-year-old Iranian woman who developed a simultaneous extra -and intrauterine pregnancy after the induction of ovulation with Clomiphene. In this case, there was a delay in the detection of the ectopic pregnancy component resulting in an emergency laparotomy being performed. Fortunately after the laparotomy, the intrauterine pregnancy was not affected and it progressed satisfactorily until 37 weeks. A healthy male baby was delivered by caesarean section.

**Conclusion:**

This case suggests that a heterotopic pregnancy must always be considered in patients presenting with pelvic pain even in a confirmed intrauterine pregnancy, particularly after the induction of ovulation by Clomiphene Citrate or assisted reproductive technology. Every clinician treating women of reproductive age should keep this diagnosis in mind. It also demonstrates that early diagnosis is essential in order to salvage the intrauterine pregnancy and avoid maternal morbidity and mortality.

## Introduction

A coexistence of an extra -and intrauterine pregnancy (IUP) is defined as a heterotopic pregnancy (HTP) [1-3]. It is a rare form of twin pregnancy, with an estimated incidence of 1/7000 to 1/30,000 in spontaneous pregnancies. It is also reported to be as high as 1% after the use of assisted reproductive technology (ART) [1,2,4,5]. Clomiphene Citrate (CC) which increases the rate of twinning could be associated with a HTP rate of 1/900 [6]. Aside from the difficulty of diagnosing the problem, management can be difficult and may be life threatening even when surgical intervention with laparotomy is performed [2].

This study describes the ruptured tubal HTP in a patient who conceived with the aid of CC, who presented at six weeks of gestation and was treated with an immediate laparotomy. The remaining course of the pregnancy was uneventful, with a caesarean section (CS) delivery of a healthy infant at 37 weeks of gestation.

## Case presentation

A 22-year-old nulliparous Iranian woman presented with 2 weeks of amenorrhea, mild lower abdominal pain, vaginal spotting, vomiting and diarrhoea. She had taken CC due to a history of 18 month's primary infertility. She was pale with a pulse rate of 100 beats/minute and blood pressure of 100/60 mmHg. Laboratory findings revealed haemoglobin of 11.2 g/dL and hematocrit of 34%. The pregnancy test was positive. Ultrasonography (USG) demonstrated the presence of a normal IUP with no other pathological signs, and no fluid effusion was reported in the pelvic cavity.

She was hospitalized and referred to the gynaecology ward for observation and conservative treatment with antiemetic and fluid replacement. Over the subsequent 24 hours, she complained of a sudden worsening of her abdominal pain and vaginal bleeding. On examination, she was tender in the lower abdomen with guarding and rebound tenderness.

**Figure 1:**
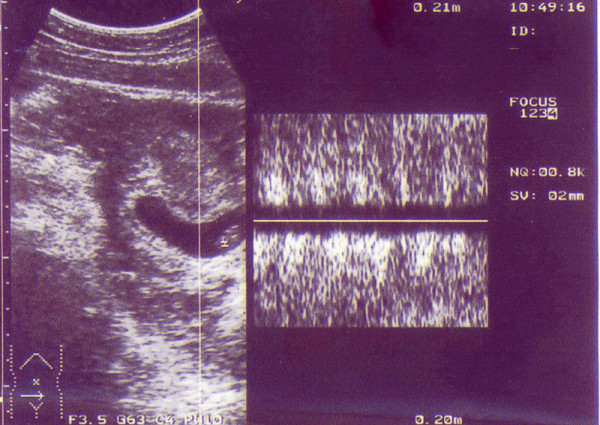
Abdominal sonogram before operation shows intrauterine gestational sac containing foetal pole with positive foetal cardiac motion with a normal spectral trace on pulse Doppler.

**Figure 2:**
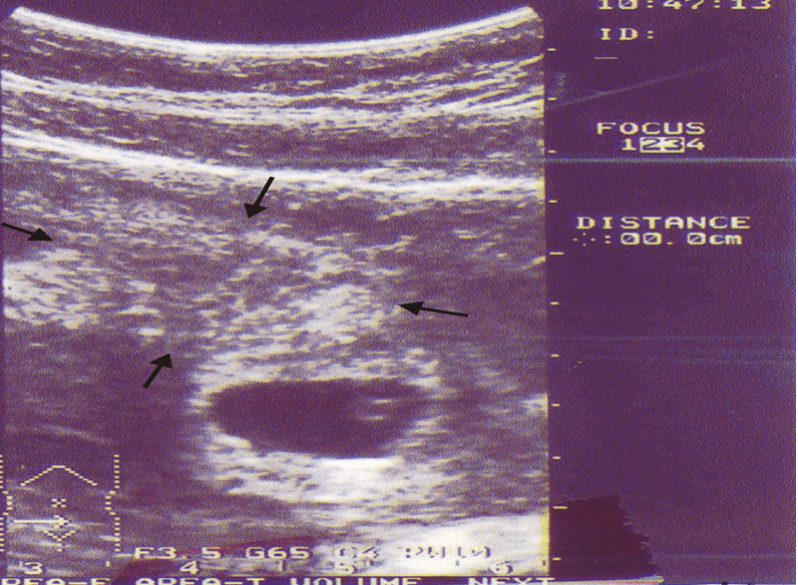
Abdominal sonogram before operation shows intrauterine gestational sac with Yolk sac and an echo complex mass in the left site of the pelvis (arrows).

A second transabdominal sonography utilizing a 3.5 MHz convex transducer was carried out by another sonologist and the results showed a well-defined foetal pole with a crown-rump length (CRL) of 18 mm equivalent to 7 weeks gestation, and yolk sac. The foetal cardiac motion was positive with a normal tracing by pulse Doppler (Figures 1 and 2).

There was also an echo complex mass in the left side of the pelvis (Figure 2). The pelvic cavity, particularly in the left lower quadrant, was full of echo complex images. The boundary of the ovaries and tubes, particularly in the left, was obscure. These findings demonstrated first an IUP with a ruptured tubal pregnancy and if not, then an IUP with a ruptured ovarian cyst.

Her haemoglobin concentration had dropped to 8.8 g/dL and hematocrit to 27%. Because of the clinical presentation, laboratory and sonographic findings, the patient was taken directly to the operating room. She was transfused with three units of whole blood. An emergency laparotomy was done under general anaesthesia that revealed 1500 mL of old blood and abundant clots and a ruptured middle left tubal pregnancy.

**Figure 3:**
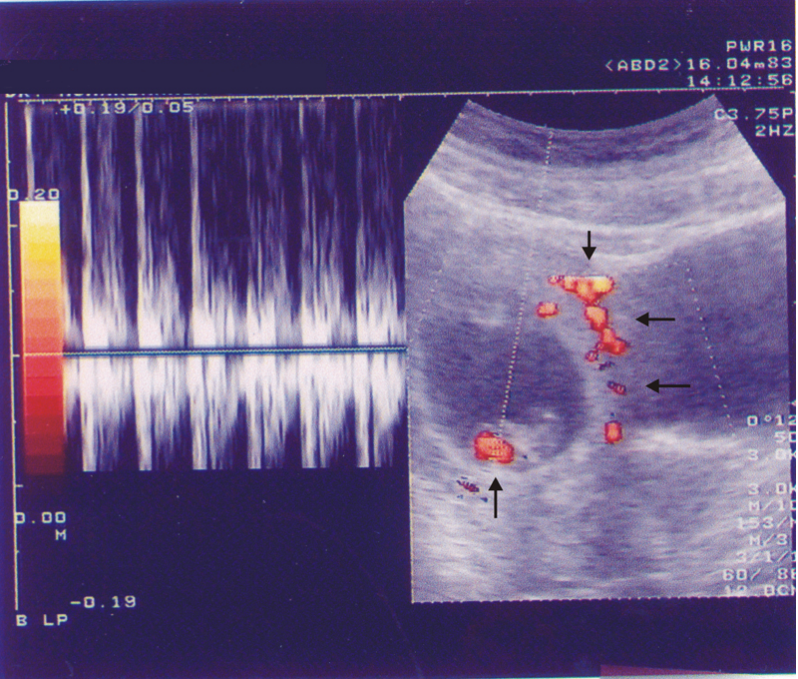
Two weeks after the operation, an abdominal sonogram shows the IUP at 9 weeks gestation and the power Doppler sonogram demonstrates colour signals at the site of the foetal heart (arrow) and retroplacental vessels (arrows).

A left salpingectomy was performed. The histopathological examination of tissue confirmed a left tubal ectopic pregnancy which was ruptured at the ampullary portion. Postoperatively her course was uneventful, and she was discharged in good general condition on the third day after the operation. Two weeks after surgery, a live IUP with a CRL equivalent to 9 weeks gestation was visualized on a transabdominal ultrasound and which also showed a marked trophoblastic flow on colour Doppler (Figure 3).

The pregnancy continued without any significant complication. She was successfully delivered of a male infant at 37 weeks gestation by CS (due to spontaneous onset of labour and contracted pelvis), the birth weight was 3100 g and her postnatal recovery was unremarkable.

## Discussion

HTP was first described by Duverney in 1708 [3,7]. Nowadays, the use of ART and fertility agents such as CC can increase a patient's risk of a HTP probably due to the combined effects of hyperstimulation and the subsequent, simultaneous transfer of several embryos into the uterus with retrograde flow into the fallopian tubes. Indeed, any factor predisposing a patient to an increased risk of ectopic pregnancy (EP) and/or multiple gestations may contribute to HTP [3,7-9]. In our patient, pregnancy also occurred in association with ovulation induction by CC.

The majority of HTP cases are diagnosed late. Significant morbidity and occasional mortality have been reported as a result of a delay in diagnosis [3]. As no single investigation can predict the presence of a HTP, it should be suspected in any patient who presents with lower abdominal pain in the early phase of an obvious IUP following fertility treatment [7,10].

Often, abdominal and pelvic USG fails to show the EP or is misinterpreted because of the awareness of an existing IUP [3,9] but demonstration of an IUP is no longer a reliable indicator for excluding an EP [3,5].

Most ultrasonographic reports make no mention of a search for coexistent EP when evaluating intrauterine gestation, because a HTP is still thought to be extremely rare and for this reason, almost all EPs are diagnosed by excluding an IUP [8].

Our case also presented early in the pregnancy with a history of nausea, scant vaginal bleeding and lower abdominal pain. These symptoms are common in IUP. There was also a delay in the detection of the EP component, therefore diagnosis was not made until an EP rupture had occurred and the patient developed haemoperitoneum and instability of her vital signs. Although the primary USG helped to confirm the presence of an IUP, it failed to identify the EP, while a HTP as a cause for abdominal pain should have been suspected immediately in our case.

The management of HTP remains controversial. Surgical therapy has been the traditional mainstay but involves surgical and anaesthetic risks to both the mother and IUP [9]. Studies suggest that laparoscopic management is preferred over laparotomy in patients with a suspected EP, and with a documented IUP because of minimal manipulation of the uterus [7].

A non-surgical approach can be used safely and effectively to manage patients who are clinically stable and where a HTP is recognized relatively early in gestation. The successful non-surgical management of six cases of HTP using potassium chloride (KCl) injection into the tubal EP has been reported [9]. In our case, if EP had been diagnosed early, then it might have been possible to complete the surgery with the laparoscope, but because of hemodynamic instability in our case, an urgent laparotomy was arranged.

## Conclusion

We can conclude that HTP must always be considered in patients presenting with abdominopelvic pain in the face of a documented IUP, because the presence of an IUP can no longer be considered reassuring and a HTP has to be ruled out. Thus, we recommend that all patients shown on USG to have an IUP should be given a comprehensive pelvic ultrasound so that the possibility of a simultaneous HTP may be excluded. We also emphasize the need for prompt and immediate action at the first sign which indicates a HTP, to avoid missing this potentially life-threatening condition.

## Consent

Written informed consent was obtained from the patient for publication of this case report and accompanying images. A copy of the written consent is available for review by the Editor-in-Chief of this journal.

## Competing interests

The authors declare that they have no competing interests.

## Authors' contributions

AH interpreted the patient's sonographic findings, suggested a heterotopic pregnancy. EK searched the literature, drafted the manuscript and revised the manuscript. SM was the surgeon of the patient (laparotomy and C/S), clinical assessor. AH, EK and SM authors read and approval the final manuscript.
